# Effects of graded calorie restriction: XXII. Impact of long-term graded calorie restriction on tissue partitioning, digestive efficiency, bone health, and coordination in male C57BL/6J mice

**DOI:** 10.1093/gerona/glaf168

**Published:** 2025-09-19

**Authors:** Sharon E Mitchell, Lucile Heib, Cara L Green, Davina Derous, Catherine Hambly, John R Speakman

**Affiliations:** School of Biological Sciences, University of Aberdeen, Aberdeen, Scotland, AB24 2TZ, United Kingdom; School of Biological Sciences, University of Aberdeen, Aberdeen, Scotland, AB24 2TZ, United Kingdom; School of Biological Sciences, University of Aberdeen, Aberdeen, Scotland, AB24 2TZ, United Kingdom; School of Biological Sciences, University of Aberdeen, Aberdeen, Scotland, AB24 2TZ, United Kingdom; School of Biological Sciences, University of Aberdeen, Aberdeen, Scotland, AB24 2TZ, United Kingdom; School of Biological Sciences, University of Aberdeen, Aberdeen, Scotland, AB24 2TZ, United Kingdom; Shenzhen Key Laboratory of Metabolic Health, Center for Energy Metabolism and Reproduction, Shenzhen Institutes of Advanced Technology, Chinese Academy of Sciences, Shenzhen, 518055, People's Republic of China; State Key Laboratory of Molecular Developmental Biology, Institute of Genetics and Developmental Biology, Chinese Academy of Sciences, Beijing 100101, People's Republic of China; Institute of Health Sciences, China Medical University, Shenyang, Liaoning province 110122, China

**Keywords:** Calorie restriction, Bone health, Neurodegeneration

## Abstract

Calorie restriction (CR) is the reduction in calorie intake while avoiding malnutrition. CR increases longevity and attenuates the development of many age-related diseases, although some unfavorable responses have been reported. In response to CR, energy is withdrawn from tissues to correct the energy deficit. Changes in tissue mass over short-term, 3 months graded CR (STCR) were complex and while most tissues reduced size, some grew. Employing a graded long-term CR (LTCR) protocol in male C57BL/6J mice, tissue utilization, digestive efficiency, bone health and motor coordination was investigated. Mice were restricted by 10%-40% over 580 days/19 months and sacrificed at 24-month old. Control mice fed *ad libitum* in the 12-hr darkphase only (12AL) were regarded as 0CR. The patterns of tissue weights, digestive efficiency, and bone measurements across the levels of CR were consistent between the STCR and LTCR studies, highlighting shared similarities over both experiments. Notable differences were enhanced utilization of the reproductive accessory organs which could be linked to a shutdown of the reproductive axis; reduced utilization of the spleen, changes in the hierarchy of investment in the digestive organs which was not linked to digestive efficiency. The vital organs were protected from utilization, with preservation of the brain by CR, presumably linked to reduced neurodegeneration and sustained coordination. The favorable effects of LTCR on bone health contradict previous negative reports. Overall, morphological changes determined within 3 months of CR, persisted to 19 months. The pattern of tissue utilization may be critical to the beneficial effects of CR.

## Introduction

The health benefits and extension to longevity in response to calorie restriction (CR) has been well documented in numerous taxa^1–3^ with promising findings also reported in non-human primates^4^ and humans.^5^ CR is a dietary intervention where total food intake is restricted while adequate nutrition is provided, however, exactly how CR elicits these beneficial responses remain unclear.^6^ Although, the majority of published studies report the level of CR, up to 65%, to be linearly related to the extension of lifespan^7–9^ some reports contradict this.^10^ Exploiting this relationship between CR level and lifespan, we propose that mechanisms generating the beneficial effects of CR would also likely follow a linear response. Over a series of previous papers, we have utilized a protocol of graded levels of CR, from 10% to 40%, to characterize the short-term (84 days/3 months) response to CR (STCR).^11–14^ Here, we investigate the effects of long-term (580 days/19 months) graded CR (LTCR).

CR results in an energy deficit and a disruption of energy balance which must be immediately addressed.^15^ Under CR, energy intake cannot be increased therefore, to balance the energy budget energy expenditure may be reduced. In the STCR study we found mice lowered body temperature.^12^ In addition, energy can be withdrawn from tissues, particularly fat stores, and digestive efficiency may be increased.^11^ Longevity is associated with body size and notably, within species it is the smaller individuals that survive the longest^16^ which contrasts between species comparisons, where larger species live longer.^17^^,^^18^

Body composition changes in response to CR are more complex than a simple loss of fat and our STCR study indicated that male C57BL/6J mice responded to the energy deficit by complex and tissue specific responses, appropriately allocating energy for tissue utilization but also for investment.^11^ All fat tissue, but particularly visceral, was utilized for provision of energy.^11^ The utilization of white adipose tissue was related to hypotrophy.^19^ Vital organs such as the liver and brain (but also testes and tail) appeared protected from utilization while tissues comprising the digestive tract actually increased in size, that is, energy was deposited, and these organs were invested in.^11^ This increase in digestive tract has been potentially linked to the gorging of the food observed in mice under CR^20^^,^^21^ and the need to maximize the energy assimilation.^22^ The mechanisms that govern exactly which tissues energy is deposited into, or withdrawn from, are poorly understood, yet have profound impacts. Body composition changes have direct implications for metabolic and hormone profiles, glucose/insulin homeostasis, and resting energy expenditure. The pattern of tissue partitioning following CR may be critical to the beneficial effects of CR as it may influence later disease risk, including obesity and type 2 diabetes.

An interplay must exist between the tissues determining from which energy is withdrawn or deposited. The regulation of energy balance is centrally controlled, with the brain responding to a multitude of internal and external signals of energy availability. In the STCR we found correlations between body composition and various circulating hormones such as leptin, insulin, and IGF-1^23^ which are potentially linked to lifespan.^24^^,^^25^ In our STCR study, we found that although the brain was relatively protected, it was still progressively utilized at higher levels of restriction. The long-term consequences of this however are unclear. Improvements in brain health were also associated with CR owing to an enhanced effect on cognitive functions or retarded effect on the progression of neurodegenerative diseases.^26^ In rhesus macaques CR slowed age-related sarcopenia, hearing loss, and brain atrophy in several subcortical regions.^27^

How LTCR impacts age-related body composition changes, such as muscle loss sarcopenia, is important. Sarcopenia has multiple negative health implications and is a major risk factor for mortality in later life.^28^ In addition to sarcopenia, osteopenia, osteoporosis, and lipopenia are characteristics of old age which have been shown to be ameliorated by CR.^29^ However, alongside the positive effects of CR are reports of several negative health effects including hunger,^13^ decreased lung capacity and risk of developing emphysema,^30^ and bone loss.^31^^,^^32^

The current article presents comprehensive analysis of tissue partitioning, bone health, digestive efficiency, cognitive abilities, and motor coordination in response to graded levels of LTCR in C57BL/6J male mice, initiated at 140 days/5 months, equivalent to young adult in human terms.

## Methods

### Animals

For a detailed description of the study protocol please refer to.^33^ All procedures were compliant with the Animals (Scientific Procedures) Act 1986 in accordance with the ARRIVE Guidelines.^34^ CR was initiated in 64 male C57BL/6J (Charles River, Ormiston, UK), at 140 days/5 months of age. Predetermined humane endpoints led to the euthanasia of 31 mice over the 588 days/19 months of CR. Survival and body mass dynamics of these mice were previously reported which includes details of necroscopy findings (Tables S1 and S2^33^)

Mice were single housed with *ad libitum access* to water. Food was provided at the start of the dark phase and removed at the start of the light phase, that is, a 12-hr *ad libitum* (12AL) feeding regime. Control mice remained on the 12AL feeding (*n* = 14). Four graded CR groups were restricted by 10%-40%, that is, fed 10%-40% less than their individual total daily calories measured at baseline. 10CR (*n* = 14), 20CR (*n* = 12), 30CR (*n* = 12), and 40CR (*n* = 12). All mice were fed D12450H over baseline (20% protein, 10% fat, and 70% carbohydrate of which 17% was sucrose; Research Diets, USA). Over the CR phase 12AL, 10CR, & 20CR continued to be fed D12450H while 30CR and 40CR mice were fed D13020504 which was matched by nutrients to D12450H but contained a 40% increase in the vitamins mix to prevent malnourishment.

### Dissection

The remaining 33 mice were sacrificed at 720 days/24 months of age by CO_2_ overdose. Blood was sampled by cardiac puncture, organs rapidly removed, weighed, and immediately snap frozen in liquid nitrogen. Final numbers were 12AL (*n* = 5), 10CR (*n* = 4), 20CR (*n* = 6), 30CR (*n* = 8), and 40CR (*n* = 10). Twenty-two tissues were dissected: vital organs (brain, liver, heart, kidney, lung, pancreas, and spleen); structural organs (carcass, skin, tail); adipose tissue (epididymal, subcutaneous, retroperitoneal, mesenteric, omental, and brown adipose tissue); alimentary tract organs cleared of content (stomach, small intestine, caecum, and colon); reproductive accessory organs which included the penis, seminal vesicles and vas deferens, and testes which were weighed separately. The right leg was wrapped in tissue soaked in phosphate-buffered saline, sealed in a plastic bag, and stored at −20 °C for measurements. The left leg was stored in glutaraldehyde at −4 °C for analysis of microarchitecture using microcomputed tomography scanner (microCT; details provided below).

### Digestive efficiency

Feces were collected over 6-8 days of baseline and the final week of CR. Feces were weighed and dried at 60 °C for 14 days. The gross energy (GE) content for feces, plus the 2 diets were measured using the semi-micro 1109 oxygen bomb calorimeter (Parr 6200 calorimeter, Scientific & Medical Products Ltd, UK). Energy assimilated (EA kJ/day) is the difference in energy consumed and that lost through feces. Urinary loss was assumed to be ∼3% loss. The digestive efficiency is the percentage of the ingested energy that is assimilated as measured by apparent energy assimilation efficiency (AEAE) % and calculated as follows:

$$\mathrm{AEAE}\;(\%)\;=\frac{\;\mathrm{GE}\;\mathrm{Food}\;\mathrm{intake}\;-\mathrm{GE}\;\mathrm{Feces}}{\mathrm{GE}\;\mathrm{Food}\;\mathrm{intake}}*100$$

### Bone characteristics

Bone mineral density and content was measured by Dual energy x-ray absorptiometry analysis (DXA) at 8 timepoints; 0, 28, 56, 84, 168, 252, 365, 580 days (0, 1, 2, 3, 6, 9, 12, and 19 months) of CR. Mice were anesthetized with isoflurane for the duration of the scan. Data was corrected using an equation specific for the DXA machine.^35^

Bones (tibia and femur) were carefully cleaned, of all muscles and ligaments. The length and diameter of each bone was measured using a digital micrometer (±0.01 mm, RS 572-044, Mituyoyo, Andover, UK). Each dimension was measured three times by 3D microCT (Skyscan 1072 X-ray Microtomograph Scanner; Skyscan, Aartselaar, Belgium). Skyscan NRecon software was used to reconstruct the images to 3D. Images were analyzed using CTAN software. The fractional bone volume relative to the total volume (BV/TV%), trabecular thickness (Tb.Th µm), trabecular separation (Tb.Sp µm), trabecular number (Tb.N mm^−1^), trabecular pattern factor (Tb.Pf mm^−1^), the structural model index (SMI), and the degree of anisotropy (DoA) were recorded. For detailed description of methodology, please refer to.^36^

### Coordination and neurodegenerative tests

Motor function and neurodegenerative problems were determined in the final 2 weeks of CR using 4 specific tests.^37^ Each test was performed 4 times and scored on a scale from 0 to 3 with 0 representing an absence of relevant phenotype and 3 an abnormal phenotype. (1) The ledge test measured the coordination and balance of the mice. Losing balance or falling off the ledge scored higher. (2) Hindlimb clasping was used as a marker of disease progression such as neurodegeneration. Clasping both legs scored 3 and was considered abnormal. (3) Gait test was used as a marker of co-ordination and muscle function. Difficulty moving and supporting body weight scored 3. (4) Kyphosis, the dorsal curvature of the spine was also analyzed. The inability to straighten spine scored 3.

### Statistical analysis

All analyzes were performed using R Statistical Software (v4.1.1^38^) The ggplot2 R package was used for data analysis and visualization.^39^ Tissue partitioning was examined by fitting a linear least square fit regression to the logged dissected tissue weights plotted against logged cull body mass. The gradient (*β*) was used to express the changes in tissue weights. *β* = 1 indicated changes in tissues were proportional to the body mass. *β* > 1 would indicate tissues were preferentially utilized. *β* = 0 to 1 would indicate protection from change and *β* < 0 indicated growth of the tissue, that is, investment.

Hierarchical clustering was based on the Pearson correlation coefficients of the dissected tissues. Clusters were based on the Euclidean method and correlation distance was based on complete linkage clustering. Data was manipulated using the gdata R package. The correlation matrix was generated using corrplot R package^40^ and represented by a heat map (value from −1 to 1).

The level of CR was treated as a continuous variable (0–40, with 0 representing the 12AL controls). Linear models were fitted to predict tissue change and bone characteristics over graded levels of CR (formula: tissue ∼ Level). Baseline body mass (BL_BM) was added as a covariate to explain whether changes were impacted by body mass (formula: tissue ∼ Level + BL_BM). To explore changes in digestive efficiency (2 timepoints) or bone mineral density/content measured by DXA (8 timepoints) in response to graded levels of CR over time a linear mixed model (estimated using REML and nloptwrap optimizer) was fitted. Time and level of CR were both treated as continuous variables and included as covariates with mouse ID as a random effect. Standardized parameters were obtained by fitting the model on a standardized version of the dataset. 95% Confidence intervals and *P*-values were computed using a Wald *t*-distribution approximation. A linear model (estimated using OLS) was fitted to determine whether changes in AEAE was linked to the morphology of the digestive tract. Poisson regression was used to analyze the scores from the 4 neurodegenerative tests.

To assess the relative rate of change with CR between the LTCR and the STCR studies, the gradient of the effect of CR level on any given trait were obtained and statistically compared between the two studies using the lsmeans package.^41^ Using this method allowed for a direct comparison between the two studies and determined if any differences in effect of CR occurred.

## Results

### Tissue partitioning

**Figure 1.**
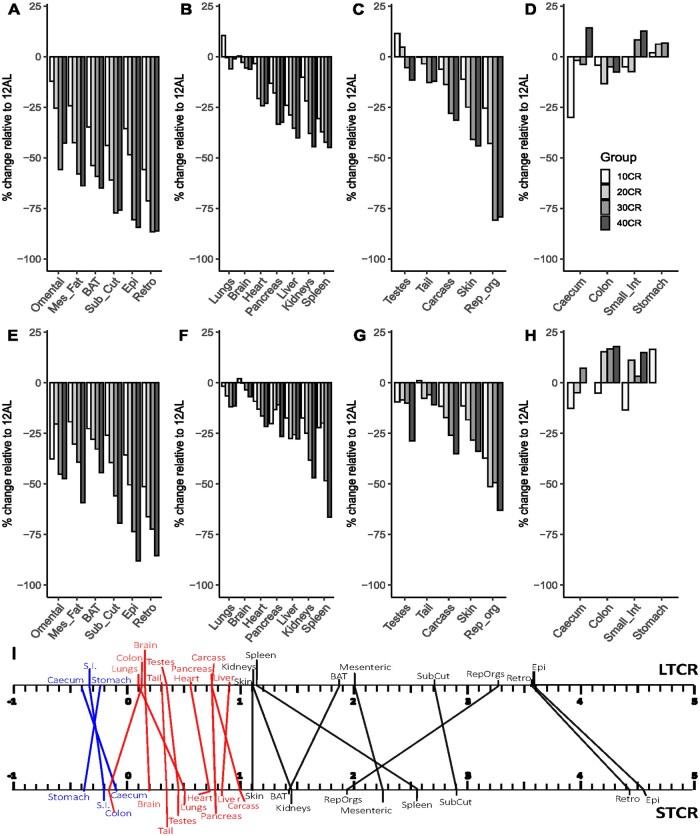
Changes in tissue size in response to long term calorie restriction (LTCR, 588 days/19 months, A-D) and short term calorie restriction (STCR, 85 days/3 months, E-H). Control mice were fed ad libitum for 12 hr (12AL). CR groups were fed 10%, 20%, 30%, and 40% less their baseline food intake (10CR, 20CR, 30CR, 40CR). Percentage changes in mass of each organ are shown relative to 12AL. (A, E) adipose tissue depots, (B, F) vital organs, (C, G) structural and reproductive organs, (D, H) digestive organs. (I) Tissue partitioning. Bottom panel: STCR, Top panel: LTCR. A gradient of <0 indicated investment (shown in blue), 0-1 protection (shown in red), and >1 utilization (shown in black). Data for STCR taken from.^11^ See Figure S1 for the log plots of each tissue plotted over levels of CR for both STCR and LTCR. BAT, brown adipose tissue.

In response to LTCR, energy was differentially utilized from the tissues (Figure 1A-E). The majority of tissues followed a graded pattern of loss of mass corresponding with the level of CR. Linear models were fitted to predict tissue mass over the graded levels of CR (0 to 40). Of the 22 tissues analyzed the linear model was significant in all tissues except the lungs, spleen, and the organs of the digestive tract (refer to Table S1). The inclusion of baseline body mass as a covariate did not impact the model (refer to Table S1). While the majority of tissues reduced in mass with increasing level of CR some tissues gained mass that is, caecum, small intestine, and stomach (Table S1). The percentage of reduction in mass relative to the 12AL group was maximal in the visceral fat depots, that is, epididymal and retroperitoneal fat depots were preferentially utilized (−86% and −84%, respectively, at 40CR relative to 12AL). The remaining fat depots subcutaneous (−76%), BAT (−65%), and mesenteric (−64%) also provided a high energy source (Figure 1A). Of the vital organs, spleen, kidneys, and liver were reduced relative to 12AL following LTCR (averaging ∼ −43% at 40CR; Figure 1B). Change in heart mass was similar, ∼23% loss, over 20CR, 30CR, and 40CR (Figure 1B). Brain mass change was minimal, from −0.3% to −6% over the restriction levels. Pattern of mass change in the lungs ranged from a 10% gain in mass at 10CR and a −6% reduction at 30CR (Figure 1B). The reduction in reproductive accessory organs was comparable to the visceral fat tissues with a ∼80% loss at high levels of CR. Testes, conversely, grew at 10CR and 20CR with a −11% loss found at 40CR (Figure 1C). The structural organs, carcass, and skin, both followed a graded reduction (Figure 1C). Tissue partitioning was heterogeneous among the digestive organs with growth in 3 of the 4 digestive organs observed (Figure 1D). Over 10 to 40CR, the stomach grew 2%-21%. Although a gain in mass of small intestine (13%) and caecum (14%) was measured at 40CR, losses were recorded at lower CR levels. The change in colon mass over graded CR was not significant (Figure 1D). These patterns of change were similar to that found in the previously reported STCR study (Figure 1E-H).^11^

Using the gradient (*β*) from fitted linear least square regression of the logged dissected tissue weights plotted against logged cull body mass, tissue partitioning was visualized (­Figure S1). Tissue partitioning data from the LTCR study was compared with previously published data from STCR^11^ (Figure 1I). A gradient of 1 would indicate the tissue changed relative to the body mass. Tissues were, therefore, divided into protected (*β* = 0-1), invested, that is, grew (*β* < 0) and preferentially utilized (*β* > 1) (Figure 1I). A remarkable similarity in tissue partitioning was found between the studies despite the 16-month difference in duration of CR (Figure S1 and Figure 1I). In the STCR study, 4 tissues were invested (digestive tract), 7 protected (mainly vital organs), and 10 utilized (mainly adipose and structural).^11^ There were only 2 changes in the allocation of tissues to these three groupings following LTCR: 3 invested, 9 utilized, and 9 protected.

The patterns of tissue weights were consistent between the LTCR and STCR studies (Figure S1) and no significant differences were found in the effect of CR level in 17 out of the 21 tissues (*P* > .05, Table S2). Of the 4 tissues that showed a significant difference between the STCR and the LTCR, 2 tissues were more utilized in the LTCR compared to STCR (BAT and reproductive organs), 1 tissue was less utilized in the LTCR compared to the STCR (spleen), and 1 tissue was protected (colon) in the LTCR study while invested in in the STCR.

In both studies, epididymal and retroperitoneal were preferentially utilized, albeit to a lesser extent in the LTCR. The reproductive accessory organs were the third most utilized tissue under LTCR, increasing utilization from *β* = 1.94 in the STCR study to 3.29 in the LTC (*t*_68_ = −2.97, *P* = .004). BAT utilization also higher in the LTCR study (*t*_69_ = −2.27, *P* = .03). Conversely, utilization of the spleen was reduced over the longer CR duration (*β* = 2.55 vs 1.16, *t*_69_ = 2.50, *P* = .01). The effect of CR level on remaining utilized organs matched that of the STCR although the carcass, which was minimally utilized in the STCR study (*β* = 1.05) was fitted to the protected organs under the LTCR protocol (*β* = 0.75, *t*_69_ = 0.24, *P* = .81). A further addition to protected organs was the colon which was invested in the STCR study (*β* = −0.17 vs 0.12, *t*_69_ = −2.66, *P* = .01). The majority of protected tissues were vital organs (liver, pancreas, heart, brain, and lungs), plus the tail and testes, which was identical to the STCR. As with the STCR, digestive organs were invested in, with the exception of the colon noted above. The organ found to be most highly invested in the LTCR study was the caecum, *β* = −0.1 vs −0.4). During STCR the stomach was highest invested (Figure 1I).

**Figure 2.**
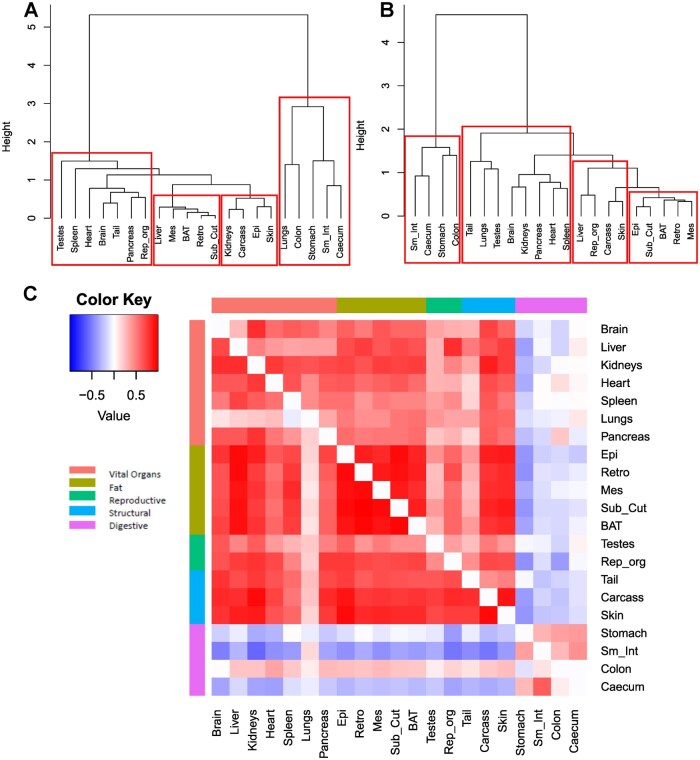
The hierarchical relationship between dissected tissues from male C57BL/6J mice undergoing (A) long-term 19 month calorie restriction (LTCR) or (B) short-term 3 month CR (STCR). Height indicates the distance between the tissues. Red boxes indicated groups of tissues most closely related. (C) Heatmap of the correlation matrix of tissue partitioning in response to STCR or LTCR. The upper triangle represents animals under STCR and the lower triangle animals under LTCR. Colored groupings at sides represents tissue functional groups. The color key for the correlations is shown at the top. Increasing intensity of red indicates a greater positive correlation and increasing intensity of blue a greater negative correlationBAT, interscapular brown adipose tissue; Epi, epididymal; Mes, mesenteric white adipose tissue; Rep_org, reproductive organs; Retro, retroperitoneal; Sub_Cut, subcutaneous fat; Sm_Int, small intestines.

Using hierarchical clustering based on Pearson correlation patterns, both the LTCR and STCR^11^ dissected tissues can be interpreted as fitting to four key clusters, however, some subtle differences existed between the studies in tissues contained within the clusters (Figure 2A and B). While the four organs of the digestive tract clustered in both studies, the lungs of the LTCR mice were also placed within this group, presumably due to growth in the 10CR mice. A second cluster consisted of the adipose tissues. All five adipose tissues grouped in the STCR with the inclusion of the liver and exclusion of epididymal fat in the LTCR. The third, and largest group, consisted mainly the protected tissues, that is, the vital organs along with the testes and tail. The fourth group was made up functionally unrelated tissues including structural organs in both studies, kidneys and epididymal fat in LTCR, liver and reproductive accessory organs in STCR.

Figure 2C illustrates the correlation coefficients of different tissues responses to CR across all the individuals in the STCR (top triangle) and LTCR (bottom triangle) studies. Despite the 16 months difference in duration of CR the matrixes are strongly matched. The components of the digestive system immediately standout as having a negative correlation with majority of other organs. This is strongest in the stomach of the STCR and small intestine of the LTCR. Of note the response of the colon to LTCR is weakly correlated to the other tissues. Apart from the lungs the remaining organs in the LTCR were strongly correlated with each other (Figure 2C).

### Bone characteristics

The effect of 19 months of graded CR on changes in bone mineral density or content (as measured by DXA) was not significant (linear mixed model: Time × Level interaction: *P* > .05). However, changes in bone mineral content were related to time alone and not the level of CR (Time, *t* = 4.74, *P* < .0001; Level, *t* = −0.49, *P* = .62). Bone mineral content rose from day 168 onwards (Figure S2). No difference in bone mineral density was found between the STCR and LTCR studies at BL or 3 months/84 days (comparison of the gradient of the effect of CR level: *t*_69_ = 0.97, *P* = .33, and *t*_67_ = −1.04, *P* = .30, respectively; Table S2). Bone mineral content did not differ between studies at BL (*t*_69_ = 0.306, *P* = .76) but following 3 months CR (end of STCR) DXA measurements were higher for the LTCR (19 months CR; *t*_67_ = −2.31, *P* = .02; Table S2).

**Figure 3.**
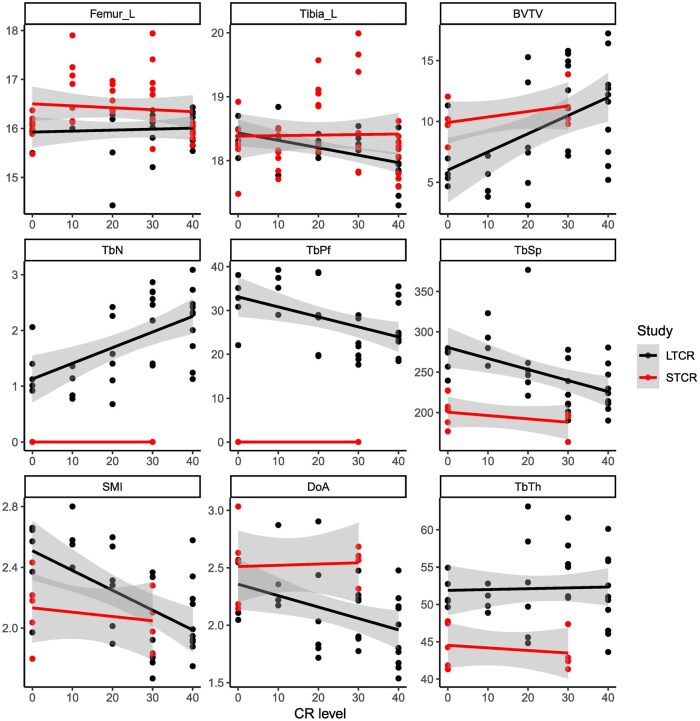
Bone characteristics from male C57BL/6J mice which have undergone long or short term graded calorie restriction (LTCR, 588 days/19 months or STCR, 85/3 months). Mice were restricted from 10% to 40%. Control mice were fed ad libitum for 12 hr per day and are represented by 0 with 10-40 for CR levels of CR (A) Femur length (mm), (B) Tibia length (mm), (C) fractional bone volume BV/TV%, (D) Trabecular Number (TbN), (E) Trabecular pattern factor (TbPf) (F) Trabecular Separation (TbSp), (G) Structural model index (SMI), (H) Degree of anisotropy (DoA), and (I) trabecular thickness (TbTh), C-I relate to Tibia. Fitted linear models are shown CR, calorie restriction.

To investigate whether CR negatively impacts bone health, a general linear model was fitted with CR level added as a covariate. Similarities between the studies were found in all measures (*P* > .05, Figure 3A-I and Table S2). While the length of the femur was not affected by LTCR (*F*_1,28_ = 0.14, *P* = .71, *R*^2^ = −0.03; Figure 3A), the length of the tibia decreased in size in relation to the level of CR (*F*_1,31_ = 10.21 *P* = .003, *R*^2^ = 0.22; Figure 3B). Following 19 months, the tibias of 40CR mice were 0.48 mm (2.6%) shorter than 12AL (Figure 3B). Cull body mass or carcass weight were nonsignificant when added as covariates (*t* = −0.13, *P* = .90 and *t* = 0.26, *P* = .79, respectively). The diameter of neither bones was affected by CR (*P* > .05).

Trabecular bone is a predictor of bone strength and higher levels of CR, resulted in a higher fractional bone volume (BV/TV%) (*F*_1, 31_=10.41, *P* = .003; Figure 3C) and a higher trabecular number (Tb.N) (*F*_1, 31_=14.85, *P* = .0005; Figure 3D). Trabecular pattern factor (Tb.Pf) and trabecular separation (Tb.Sp), indications of increased competence connectivity and separation of trabeculae, were significantly lower with higher levels of CR (*F*_1, 31_=8.50, *P* = .006 and 10.04, *P* = .003, respectively; Figure 3E and F). The structure model index (SMI) and degree of anisotropy (DoA) were also inversely related to increasing CR (*F*_1, 31_=14.79, *P* = .0006, and 6.63, *P* = .01, respectively; ­Figure 3G and H). Trabecular thickness (Tb.Th) was not affected by 19 months of graded CR (*F*_1, 31_ = 0.03, *P* = .85; Figure 3I). Overall the trabecular bone measures were indicative of improvements in bone health in the higher CR groups.

### Digestive efficiency

Components of digestive efficiency did not differ between studies at BL or the end of the study period, that is, 3 months CR versus 19 months (*P* > .05, Table S2 and Figure S3). Feces output (dry g/day) between baseline and the end of study (19 months CR) was reduced linearly with graded CR (Time by level interaction: *t* = −4.32, *P* = .0001). No change in feces output was noted in the 12AL; Figure S3A). Similarly, GE from feces and energy assimilated (EA) remained unchanged in the 12AL but were also linearly reduced over the 19 months of study in the CR groups (GE (KJ/day): *t* = −5.88, *P* < .0001, and EA (KJ/day): *t* = −2.59, *P* = .012; Figure S3B and S3C, respectively). These changes resulted in an overall decrease in apparent energy absorption efficiency (AEAE%) in the 12AL. AEAE% was maintained in the CR groups over the 19 months of CR (Time by level interaction, AEAE%: *t* = 2.5, *P* = .015) (Figure S3D). The remodeling of the digestive tract was not related to AEAE% at the end of study.

### Coordination and neurodegenerative tests

**Figure 4.**
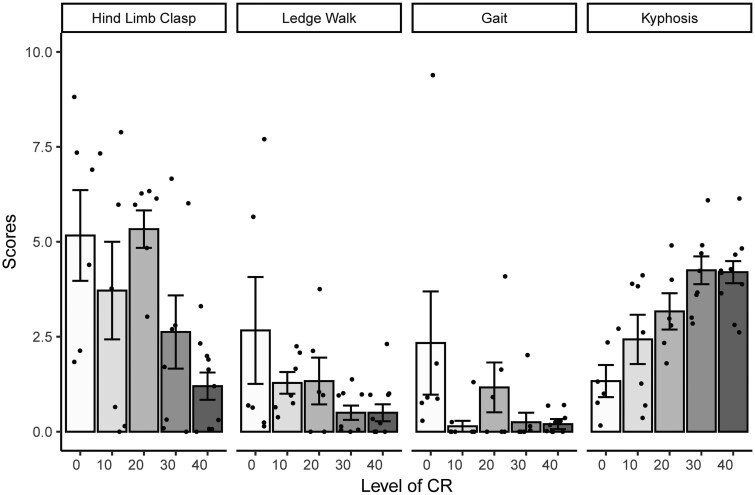
Phenotypic scoring of tests specific to motor function and neurodegeneration. Tests were carried out in the final 2 weeks of calorie restriction ∼720 days/24-month old. Mice were fed 12 hr ad libitum (12AL), or restricted by 10%, 20%, 30%, or 40% of individual baseline intake (0, 10, 20, 30, and 40, respectively) for 555 days. Hindlimb clasping is a marker of neurodegeneration, the ledge walk test measured co-ordination, the gait test is a marker of co-ordination and muscle function, and kyphosis indicates spinal curvature. Each test was performed 4 times and scored on a scale from 0 to 3 with 0 representing an absence of relevant phenotype and 3 the maximum phenotype, that is, the higher the score the more unfavorable. CR, calorie restriction.

LTCR initiated at 5 months of age positively affected measures of motor coordination measured at age 24 months (Figure 4). Animals were scored from 0 to 3 and a higher score indicates deterioration in motor function and neurodegeneration: Poisson regression; hindlimb clasping, *Z* = −4.48, *P* < .0001, ledge walking, *Z* = −3.81, *P* = .0001, and gait, *Z* = −3.7, *P* = .0002 (Figure 4). The scores for these tests were significantly reduced across CR level implying improvements. In contrast, kyphosis, an abnormality in the curvature of the spine, was increased as CR increased (*Z* = 3.49, *P* = .0005).

## Discussion

While CR is well documented to improve healthspan and extend lifespan in many species, the exact mechanisms involved in this effect are yet to be defined. Ageing is a multifactorial process, as such, so is the CR response, with CR attenuating many of the negative effects imposed by ageing, for example, cognitive decline and sarcopenia.^26^^,^^42^ Albeit seemingly contradictory, lifespan extension appears positively related to the level of CR, that is, less food led to longer life.^7–9^ However, some studies disagree.^10^ Results from the NIA study supported an improved healthspan with CR at 20% and 40% but did not find a correlation with lifespan extension.^10^ The data however, from NIA fall within the cloud of data from studies across all rodents linking level of restriction to lifespan extension suggesting these observations are not incompatible with the overall trend that more restriction generally leads to greater life extension at least up to 65% restriction.^7–9^ To investigate the CR response, we utilized a graded level approach feeding mice 0%-40% restriction of individual baseline food intake (0 represents *ad libitum group*). While our previous studies reported on short-term, 3-month CR (STCR)^11^^,^^12^^,^^23^ here, we investigated long-term 19 months CR (LTCR) using the same protocol of initiation, housing and feeding with only minor differences in diet.^33^

A predominant effect of CR brought about by the reduction in energy intake is a change in body composition.^11^^,^^33^ For stability of body mass energy balance is required, that is, energy intake is equivalent to energy expenditure. Faced with restricted energy, animals under CR cannot increase their intake, therefore, must reduce their expenditure to return to energy balance. To adjust, animals may decrease physical activity, relax thermoregulation, and utilize energy from their tissues. While energy is predominantly obtained from adipose tissue, we found a differential utilization of organs under graded STCR.^11^ While the majority of organs were reduced in size in a graded manner reflective of the level of CR, some increased. Using the dissected weights of 22 tissues, we explored the response to LTCR making comparisons to the STCR study. While duration of CR is the key effect explored, the age of the mice should also be considered with an age-related functional decline of many organs.

There were remarkable similarities in how the 22 tissues were partitioned following STCR or LTCR. Key differences were found in functionally distinct organs which the discussion focuses on: the reproductive accessory organs, spleen, and organs of the digestive tract. The organ which was most significantly affected by the increased duration of CR was the reproductive accessory organs with utilization increased significantly after 19 months of CR. The disposable soma theory postulates that faced with a scarcity of food, the maintenance of bodily function would be of foremost importance and to save resources the reproductive axis would be shut down.^43^ While the increased utilization of the reproductive accessory organs implies a reduction in the importance of these organs with extended CR, contrasting this, the testes, as observed with STCR, remained protected. In female mice a higher level of reproductive performance was found following CR^44^ but as far as we are aware no such study has been carried out in males.

Agreeing with our STCR study and previous reports, the spleen was reduced in size following LTCR.^11^^,^^45^ However, the extent of utilization of the spleen was lower compared to STCR. This may be simply explained by the age-related growth of spleen which is reported to double in size from 3 months to 24 months in C57BL/6J mice.^46^ Of course, larger spleens may indicate an unfavorable pathological state of the mice and a high variation in spleen weight was observed here. While the spleen is not considered a vital organ, it is an important secondary lymphoid organ and the site of innate and adaptive immune processes. The extension to life span induced by CR may be traded-off with a loss of immune function which may involve the spleen. Structural changes with age to the spleen may reduce the effectiveness of the immune response. While we cannot comment on function, this reduction in utilization of the spleen may aid perseveration of cell mediated immunity found following CR in C57BL/6J.^11^^,^^21^^,^^45^^,^^47^^,^^48^

Although it may seem paradoxical to invest energy into organ growth when faced with restricted energy, we and others have reported growth of the digestive tract in response to CR.^11^^,^^21^^,^^45^^,^^48^ The primary function of the digestive tract is to process the food eaten into utilizable energy via digestion and absorption of nutrients. Given the reduced intake during CR, extracting as much energy as possible from the ingested food might mitigate the deficit. Several models of energy disruption, for example, lactation, cold exposure, dietary whey protein intake, as well as CR, result in remodeling of the digestive organs to adapt to food availability.^22^ The mechanism for investment in growth of the digestive tract tissues following STCR study appeared to involve hyperplasia.^19^ The stomach is the initial site for the mechanical and enzymatic breakdown of food, hence, the larger stomachs of mice on CR potentially facilitated gorging of their daily intake within a few hours.^21^ The increase in mass of the intestinal tissues may be related to their length, diameter, or thicker walls. We cannot differentiate between these from the measures we made, however, increased mass and longer intestines following 20 days of 40CR recorded in BALB/c were related to a reduction in energy excreted in feces.^48^ Despite remodeling of the digestive tract following LTCR, an increase in digestive efficiency was not apparent, but the observed decrease in AEAE found in the 12AL fed mice was avoided.

Adipose tissue has two vital physiological functions, energy storage and thermoregulation but also plays important role in immunity and protection. White adipose tissue exists in 2 structurally and functionally distinct forms: subcutaneous, beneath the skin, and visceral which lines the internal organs (epididymal, mesenteric, retroperitoneal, and omental). While both depots are important for the storage of excess calories as triglycerides, visceral adipose tissues are more readily responsive to immediate energy deficit and the subcutaneous depots, more important as an insulative/cushioning layer, are regarded as a more long-term energy store.^49^ This was evident from both the STCR and LTCR studies (Figure 1). We previously reported the changes in adipose tissue was caused solely by adipocyte hypotrophy and was proportional to the level of CR.^19^ A preferential reduction in visceral fat by CR^50^ or following surgical removal^51^ were directly linked to an extension in lifespan. Several knockout (KO) mouse models of reduced fat such as the fat cell insulin receptor (FIRKO), insulin receptor substrate-1 (IRS-1), S6 kinase-1, and growth hormone receptor knockout (GHRKO) mice also observe extended lifespans.^52^^,^^53^ Of interest are the long-lived Ames and Snell dwarf mice, classified as relatively obese, these mice have a higher proportion of subcutaneous fat than visceral.^54^

Adipose tissue gain and redistribution from subcutaneous to visceral depots, are associated with age.^55^ Visceral adipose tissue is linked to insulin resistance contributing to an increased risk to Type II diabetes. The preferential utilization of visceral adipose tissue, therefore, improves insulin sensitivity and may be linked to lifespan extension.^50^^,^^56^ The protection of fat mass was reported to be a significant predictor of lifespan in recombinant inbred ILSXISS mice.^57^ However, subcutaneous and visceral fat depots were not defined, therefore, any relationship between lifespan and fat redistribution could not be determined.^57^

While the majority of response to CR discussed here are beneficial, some studies have reported a detrimental impact on bone health in rodents^31^^,^^32^ and humans.^58^ CR provoked bone loss in rodents may be explained by the implementation of CR soon after weaning.^31^^,^^32^ To avoid detrimental effects on growth and development, we initiate CR once mice are fully developed. Age-related bone loss is a primary cause of osteoporosis leading to decreased mineralization, an increase in porosity of both trabecular and cortical bone, thus, reducing bone strength; all ultimately increasing the risk of bone fracture. Between the ages of 6 and 24 months *ad libitum* fed male C57BL/6J mice lost a total of 8% in bone mass; 60% trabecular bone volume and 12% cortical bone thickness of the tibia.^59^ Results from our STCR study contradicted the proposed negative impact on bones and a clear positive impact of CR on bone was recorded.^11^ Results reported here further support a favorable outcome on overall bone health following LTCR.

Decreases in bone mineral density and content are linked to excessive bone resorption; our results indicate ST and LTCR protected against the development of osteoporosis.^11^ Of note, no indication of bone resorption with age in the 12AL controls was found. Trabecular bone has a high turnover rate compared with the cortical bone and measurements of trabecular density and trabecular microstructure are used as a predictor of bone strength. Overall LTCR had a positive impact on the trabecular bone of the tibia with results indicating that bones were stronger with a reduced risk of fractures. Beneficial results were related to the graded CR levels. These trends in trabecular microstructure were observed in the STCR but lacked significance^11^ suggesting a positive impact of increased duration of CR; similar results were reported in C57BL/6 mice.^60^

Physical activity diminishes bone loss in humans.^61^ Mice on CR develop increased activity prior to feeding; known as food anticipatory activity (FAA). In the STCR study we proposed their bones were adapted to this activity stimulating bone growth and leading to longer and thicker bones.^11^ However, these effects were not replicated with the LTCR, and the length of tibia decreased with higher levels of CR. Others have also reported a reduction in tibia length after 1 month of 40CR which they linked to reductions in circulating leptin.^62^

Despite indications of bone health improvements, the incidence of spinal curvature known as kyphosis increased as the level of CR increased. Kyphosis is age-related and was apparent in all groups including the 12AL. Although kyphosis may impair gait, here the advancement of kyphosis with increasing CR levels did not appear to affect co-ordination. While impairments in motor health are often found with ageing, we found protective effects of LTCR. Three of our tests indicated that CR lessened these age-related concerns, enhancing or retarding age-related effect on cognitive functions and progression of neurodegenerative diseases. Age related impairments in balance, frailty and gait were improved with CR in a graded manner relative to the 12AL animals. These results strongly agree with studies in male Wistar rats.^60^ Motor and cognitive abilities of the rats were dependent on duration and time of implementation of CR. Results were most favorable following life-long (18 month) 40CR, initiated at 6 months, that is, equivalent to young adult phase.^63^ This was similar to the protocol used here and highlights the importance of age of initiation and the duration of CR in studies.

Results found here indicated the brain was the most protected organ and LTCR delayed brain ageing. The coordination and neurodegenerative tests suggested that mice under LTCR were less likely to develop cerebral disease linked to motor function and neurons. In the rhesus macaques (*Macaca mulatta*) CR also decreased the incidence of brain degeneration and preserved motor function indicating neural protection, similar to our results in the LTCR mice.^64^ However, an acceleration in grey matter atrophy was reported in grey mouse lemur (*Microcebus murinus*).^65^ Despite this, as with the mice here, the motor and cognitive capacities of the lemurs were not affected.^65^

Overall, the responses to LTCR in male C57BL/6J mice agreed with the reported health benefits. The current study was limited to male mice but the importance of sexual dimorphism in the response to CR is an important factor requiring further investigation. Specifically, many tissue features of ageing appear alleviated by LTCR. A remarkable outcome was the similarity in patterns found in the majority of measures (34 out of 38) between the STCR and LTCR studies despite a 16-month difference in CR duration. This is suggestive that shorter CR protocols could be employed when looking at these specific effects. The accumulation of visceral adipose tissue was greatly reduced, and this may underpin many health benefits such as improved insulin sensitivity. Although tissue function was not studied, the protection in size of the vital organs presumably maintained function over the lifespan of the mice. Importantly the brain appeared protected from utilization and was related to improvements in neurodegeneration and motor coordination compared to aged AL mice. The literature on bone health following CR is puzzling with physical activity, body weight, hormones and diet all impacting bone. Given CR induces body and fat mass loss, increased food anticipatory activity (FAA) and reductions in leptin and increases in adiponectin, the beneficial response in bone health reported here seems understandable, even if it conflicts with some previous reports of adverse effects.

## Supplementary Material

glaf168_Supplementary_Data
