# Supplementary material for: Effects of graded calorie restriction: XXII. Impact of long-term graded calorie restriction on tissue partitioning, digestive efficiency, bone health, and coordination in male C57BL/6J mice
Source: J Gerontol A Biol Sci Med Sci. 2025 Sep 19;80(11):glaf168. doi: 10.1093/gerona/glaf168 (PMC12501111; doi:10.1093/gerona/glaf168)
Supplement: glaf168_Supplementary_Data [file glaf168_supplementary_data.docx]

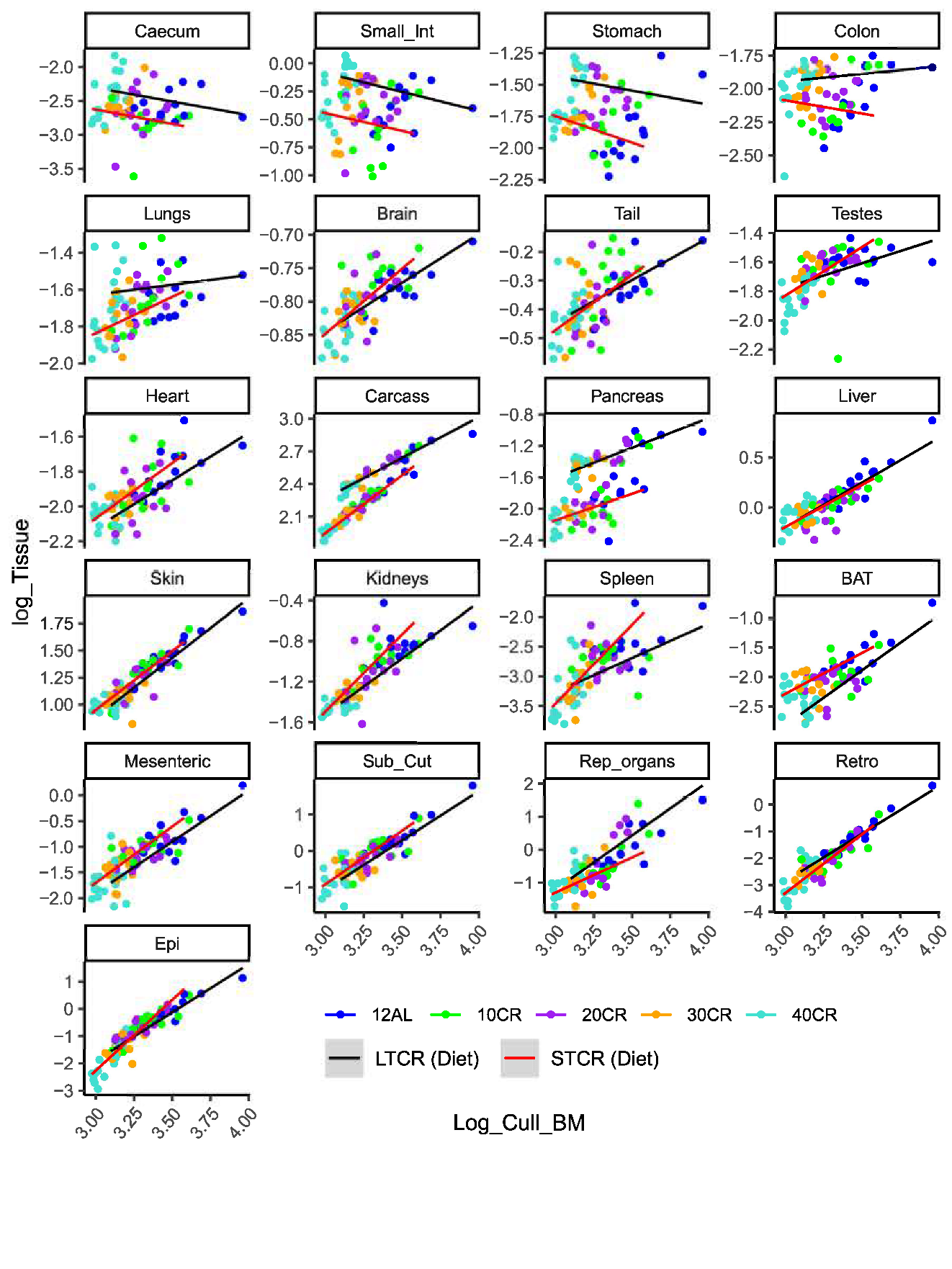
**eFigure 1 Energy partitioning of tissues in male C57BL/6J mice undergoing short or long term calorie restriction or (STCR or LTCR).** Dissected log tissue weights were plotted against cull body mass (BM) with best line fit plotted across all groups. The slope β was used to determine tissue partitioning as shown **in Figure 1E**. 12AL : mice fed 12 hrs *ad libitum*, 10CR, 20CR, 30CR and 40CR : mice restricted by 10%, 20%, 30% and 40% of their individual baseline intake.


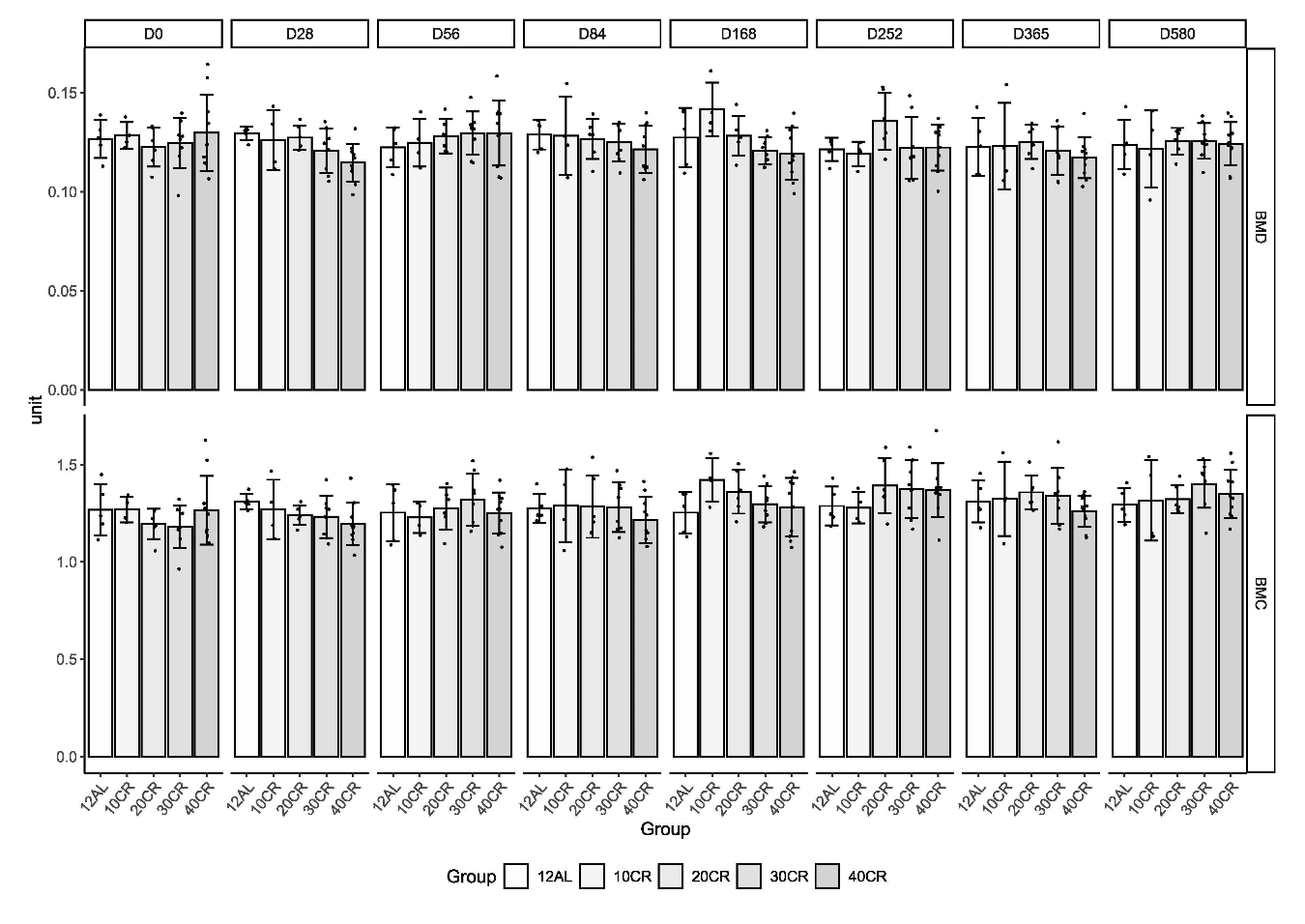


**eFigure 2 Bone mass density (BMD) and content (BMC) as measured by Dual X Ray absorptiometry** at 8 timepoints over long-term graded calorie restriction. **BMD expressed in g/cm^2^  and BMC in g.**


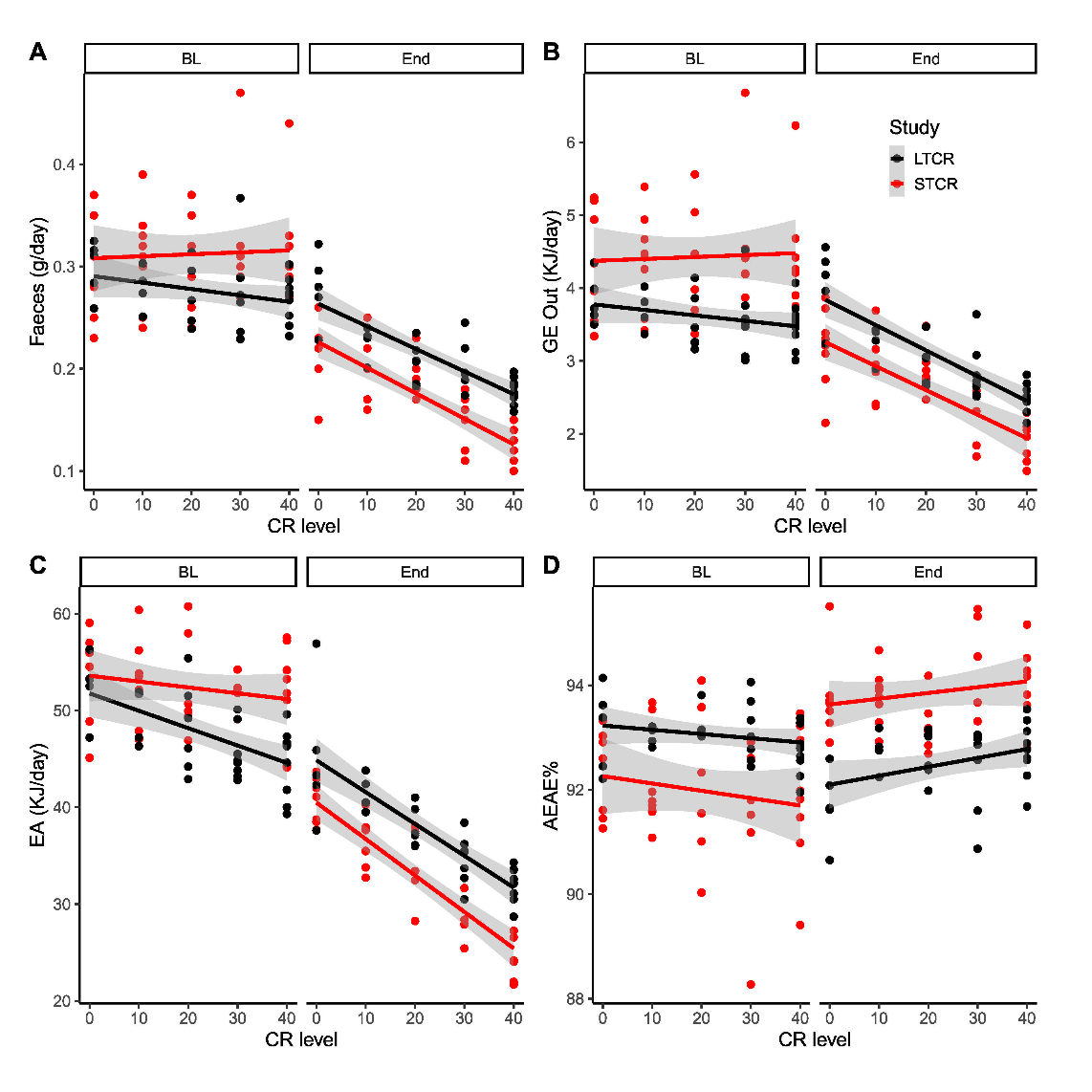


**eFigure 3 Components of digestive efficiency over short or long term graded calorie restriction (STCR, 85/3 months or LTCR, 588 days/19 months) in male C57BL/6J mice.** Faeces were collected over baseline (BL) when mice were 140 days old and at the End of study. Mice were fed 12 hours ad libitum (12AL-CR level 0), or intake was reduced by 10%, 20%, 30% or 40% of individual baseline intake. A) dry faeces weight (g/day), B) Gross energy (GE) from faeces (KJ/day), C) Assimilated energy (AE), D) Apparent energy absorption efficiency (AEAE%).

| **Tissue** | **12AL**  **(n=5)** | **10CR**  **(n=4)** | **20CR**  **(n=6)** | **30CR**  **(n=8)** | **40CR**  **(n=10)** | **F & p** | **Adj R^2^** | **CR level**  **t & p** | **BL BM**  **t & p** |
| --- | --- | --- | --- | --- | --- | --- | --- | --- | --- |
| Carcass | 15.42  ±1.63 | 14.47  ±0.84 | 13.29  ±0.94 | 11.08  ±0.70 | 10.58  ±0.44 | 71.9  <0.0001 | **0.82** | -10.78  <0.0001 | **1.91**  **0.07** |
| Skin | 4.98  ±0.95 | 4.42  ±0.71 | 3.74  ±0.43 | 2.94  ±0.33 | 2.79  ±0.23 | 41.69  <0.0001 | **0.72** | -8.35  <0.0001 | **1.13**  **0.27** |
| Tail | 0.76  ±0.06 | 0.76  ±0.04 | 0.74  ±0.02 | 0.66  ±0.04 | 0.67  ±0.03 | 16.44  <0.0001 | **0.49** | -5.12  <0.0001 | **0.99**  **0.33** |
| Brain | 0.47  ±0.01 | 0.47  ±0.01 | 0.45  ±0.02 | 0.44  ±0.01 | 0.44  ±0.01 | 14.62  <0.0001 | **0.46** | -5.00  <0.0001 | **0.52**  **0.61** |
| Liver | 1.57  ±0.49 | 1.19  ±0.12 | 1.12  ±0.07 | 1.01  ±0.06 | 0.94  ±0.10 | 26.18  <0.0001 | 0.61 | -6.17  <0.0001 | 1.84  0.08 |
| Kidneys | 0.44  ±0.05 | 0.4  ±0.03 | 0.35  ±0.04 | 0.27  ±0.04 | 0.25  ±0.01 | 72.76  <0.0001 | 0.82 | -11.06  <0.0001 | 1.41  0.17 |
| Lungs | 0.21  ±0.02 | 0.23  ±0.03 | 0.20  ±0.01 | 0.19  ±0.01 | 0.20  ±0.03 | 2.96  0.07 | 0.11 | -0.58  0.57 | 2.09  0.045 |
| Heart | 0.17  ±0.01 | 0.17  ±0.02 | 0.14  ±0.02 | 0.13  ±0.01 | 0.13  ±0.01 | 16.06  <0.0001 | 0.48 | -4.35  <0.0001 | 2.22  0.034 |
| Spleen | 0.09  ±0.04 | 0.06  ±0.02 | 0.06  ±0.01 | 0.05  ±0.02 | 0.05  ±0.02 | 4.56  0.02 | 0.18 | -2.85  0.008 | 0.12  0.90 |
| Pancreas | 0.34  ±0.02 | 0.29  ±0.04 | 0.28  ±0.03 | 0.23  ±0.03 | 0.23  ±0.03 | 24.02  <0.0001 | 0.59 | -6.68  <0.0001 | -0.15  0.88 |
| Sub Cut | 2.5  ±2.07 | 1.40  ±0.72 | 0.98  ±0.35 | 0.57  ±0.15 | 0.60  ±0.23 | 16.52  <0.0001 | 0.49 | -5.51  <0.0001 | -0.02  0.98 |
| Epi | 1.53  ±0.97 | 0.99  ±0.47 | 0.79  ±0.23 | 0.3  ±0.09 | 0.24  ±0.10 | 40.92  <0.0001 | 0.71 | -8.36  <0.0001 | 0.88  0.38 |
| Retro | 0.80  ±0.72 | 0.35  ±0.24 | 0.23  ±0.09 | 0.11  ±0.03 | 0.11  ±0.04 | 26.7  <0.0001 | 0.62 | -7.43  <0.0001 | 0.004  0.99 |
| Mes | 0.58  ±0.38 | 0.44  ±0.14 | 0.33  ±0.07 | 0.24  ±0.08 | 0.21  ±0.09 | 10.44  0.0004 | 0.37 | -4.03  <0.0004 | 0.89  0.38 |
| BAT | 0.23  ±0.14 | 0.15  ±0.06 | 0.11  ±0.03 | 0.09  ±0.03 | 0.08  ±0.01 | 17.86  <0.0001 | 0.51 | -5.37  <0.0001 | 0.96  0.34 |
| RepOrg | 2.63  ±1.27 | 1.96  ±1.39 | 1.51  ±0.81 | 0.50  ±0.28 | 0.55  ±0.22 | 22.19  <0.0001 | 0.57 | -5.73  <0.0001 | 1.59  0.122 |
| Testes | 0.19  ±0.02 | 0.22  ±0.01 | 0.20  ±0.003 | 0.18  ±0.02 | 0.17  ±0.01 | 12.65  0.0001 | 0.42 | -3.76  <0.0008 | 2.12  0.043 |
| Stomach | 0.20  ±0.05 | 0.21  ±0.01 | 0.22  ±0.02 | 0.22  ±0.03 | 0.25  ±0.02 | 3.68  0.04 | 0.14 | 2.54  0.02 | -0.184  0.85 |
| SmInt | 0.80  ±0.09 | 0.76  ±0.06 | 0.74  ±0.10 | 0.87  ±0.09 | 0.90  ±0.12 | 3.48  0.04 | 0.13 | 2.54  0.016 | 0.06  0.95 |
| Caecum | 0.09  ±0.02 | 0.06  ±0.002 | 0.09  ±0.02 | 0.09  ±0.02 | 0.10  ±0.02 | 2.17  0.132 | 0.07 | 2.07  0.047 | 0.43  0.67 |
| Colon | 0.16  ±0.01 | 0.15  ±0.022 | 0.14  ±0.013 | 0.15  ±0.014 | 0.15  ±0.010 | 1.77  0.187 | 0.05 | -0.73  0.47 | 1.45  0.16 |

**eTable 1** Tissue weights of male C57BL/6J mice following 19months of graded calorie restriction (CR) or 12 hours of ad libitum (12AL) feeding. Feeding was restricted by 10%, 20%, 30% or 40% of individual baseline food intake. Results of fitted linear model to predict tissue weight with CR level and baseline body mass (BL BM) included as a covariate. Data shown as mean±sd. Highlighted values were significant after Bonferroni correction (p<0.0022).

**eTable 2** Comparison of slopes (efigure 1) between the previous short-term (3 month) calorie restriction (CR) study and long-term study (19 month) CR study. The slopes were obtained for each of the measures and comparisons made using the lsmeans package^40^. Significant differences are shown in bold. AEAE = apparent energy assimilation efficiency. BL = baseline. End = end of study 3months CR versus 19 months CR.

| **Measure** | **Estimate** | **SE** | **df** | **t.ratio** | **P value** |
| --- | --- | --- | --- | --- | --- |
| **Tissues** |  |  |  |  |  |
| Mesenteric fat | -0.004 | 0.005 | 69 | -0.75 | 0.45 |
| **Brown adipose tissue** | **-0.009** | **0.004** | **69** | **-2.27** | **0.03** |
| Subcutaneous fat | -0.004 | 0.006 | 69 | -0.65 | 0.52 |
| Epididymal fat | 0.004 | 0.007 | 69 | 0.45 | 0.65 |
| Retroperitoneal fat | -0.001 | 0.007 | 67 | -0.14 | 0.89 |
| Lungs | 0.002 | 0.002 | 69 | 1.02 | 0.31 |
| Brain | 0.001 | 0.001 | 68 | 0.34 | 0.74 |
| Heart | -0.001 | 0.002 | 69 | -0.75 | 0.46 |
| Pancreas | -0.005 | 0.003 | 68 | -1.80 | 0.07 |
| Liver | -0.004 | 0.002 | 68 | -1.61 | 0.11 |
| Kidneys | -0.001 | 0.002 | 69 | -0.17 | 0.87 |
| **Spleen** | **0.013** | **0.005** | **69** | **2.50** | **0.01** |
| Testes | 0.002 | 0.002 | 69 | 1.21 | 0.23 |
| Tail | -0.001 | 0.001 | 69 | -0.54 | 0.59 |
| Carcass | 0.0003 | 0.001 | 69 | 0.24 | 0.81 |
| Skin | -0.005 | 0.002 | 69 | -2.60 | 0.11 |
| **Reproductive organs** | **-0.021** | **0.007** | **68** | **-2.97** | **0.004** |
| Caecum | -0.001 | 0.004 | 69 | -0.03 | 0.97 |
| **Colon** | **-0.006** | **0.002** | **69** | **-2.66** | **0.009** |
| Small intestine | -0.001 | 0.003 | 69 | -0.19 | 0.85 |
| Stomach | -0.001 | 0.002 | 69 | -0.62 | 0.54 |
| **Bones** |  |  |  |  |  |
| Bone mineral content-BL | 0.001 | 0.002 | 69 | 0.31 | 0.76 |
| Bone mineral content-End | -0.004 | 0.002 | 67 | -2.31 | 0.02 |
| Bone mineral density-BL | 0.001 | 0.001 | 69 | 0.97 | 0.33 |
| Bone mineral density-End | -0.001 | 0.001 | 67 | -1.04 | 0.30 |
| Bone volume (BV/TV%) | 0.10 | 0.089 | 38 | 1.17 | 0.25 |
| Trabecular thickness (µm) | 0.06 | 0.113 | 38 | 0.40 | 0.69 |
| Trabecular number (mm^-1^), | 0.03 | 0.014 | 38 | 2.06 | 0.05 |
| Trabecular separation (µm) | -0.95 | 0.832 | 38 | -1.14 | 0.26 |
| Trabecular pattern factor (mm^-1^) | -0.23 | 0.147 | 38 | -1.56 | 0.13 |
| Structural model index | -0.01 | 0.007 | 38 | -1.51 | 0.14 |
| Degree of anisotropy | -0.01 | 0.008 | 38 | -1.39 | 0.17 |
| Femur length (mm) | 0.006 | 0.009 | 63 | 0.66 | 0.51 |
| Tibia length (mm) | -0.01 | 0.008 | 66 | -1.54 | 0.13 |
| **Digestive efficiency** |  |  |  |  |  |
| AEAE-BL | 0.006 | 0.016 | 64 | 0.37 | 0.71 |
| AEAE-End | 0.006 | 0.012 | 62 | 0.49 | 0.62 |
| Energy assimilated (kJ/day)-BL | -0.119 | 0.069 | 64 | -1.74 | 0.09 |
| Energy assimilated-End | 0.047 | 0.053 | 62 | 0.89 | 0.38 |
| Faeces (g/day)-BL | -0.001 | 0.001 | 64 | -1.08 | 0.28 |
| Faeces (g/day)-End | 0.0013 | 0.001 | 69 | 0.69 | 0.49 |
| Gross energy out (KJ/day)-BL | -0.010 | 0.010 | 64 | -0.97 | 0.33 |
| Gross energy out-End | -0.002 | 0.007 | 61 | -0.27 | 0.79 |
